# Medicine and Art

**Published:** 2017

**Authors:** VL Purcarea

**Affiliations:** * "Carol Davila" University of Medicine and Pharmacy, Bucharest, Romania

The end of 2016 marked, among others, also an important cultural event, through its novelty and uniqueness, which took place at the oldest University of Medicine and Pharmacy in Romania. 

“Constantin Brancusi” vernissage of sculpture exhibition took place in the imposing Hall of Honor of “Carol Davila” University of Medicine and Pharmacy in Bucharest and was organized through the efforts of some culture enthusiasts, such as Constantin Barbu, Vice-president of “Mihai Eminescu” International Academy in Craiova and the famous surgeon, Prof. Mircea Beuran, MD, PhD, the President of the Senate of “Carol Davila” University of Medicine and Pharmacy in Bucharest. 

**Fig. 1:**
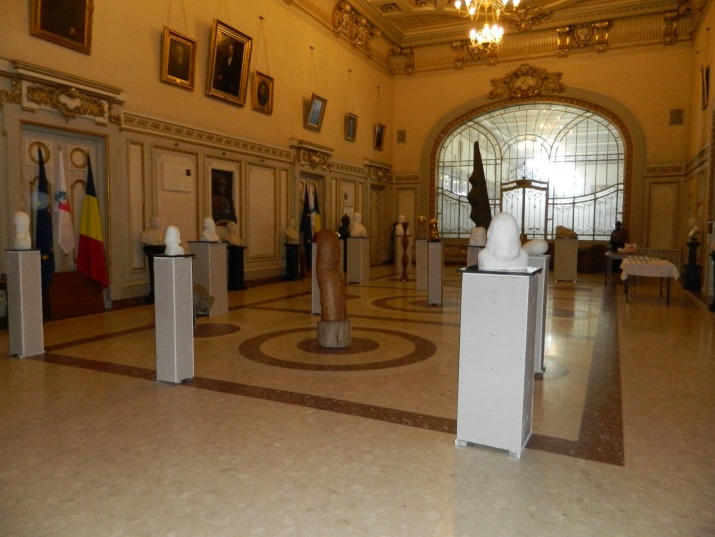
The Hall of Honor of “Carol Davila” University of Medicine and Pharmacy in Bucharest, the host of the sculpture exhibition

The exhibition presented twenty sculptures of immortal Brancusi, which were lost in the passing of time, but have been brought to life by the famous Romanian sculptor, Rodion Gheorghita. 

“Study for vanity, A muse, Old man head, The fish, The portrait of a doorman, Danaida, Red-skins, The portrait of Victoria Vaschide, The kiss, Eve, The baroness, Gheorghe Lupescu, The portrait of Daniel Poiana, etc.”, were made by master Rodion Gheorghita, through efforts which only he himself knows, having as model a picture.

**Fig. 2:**
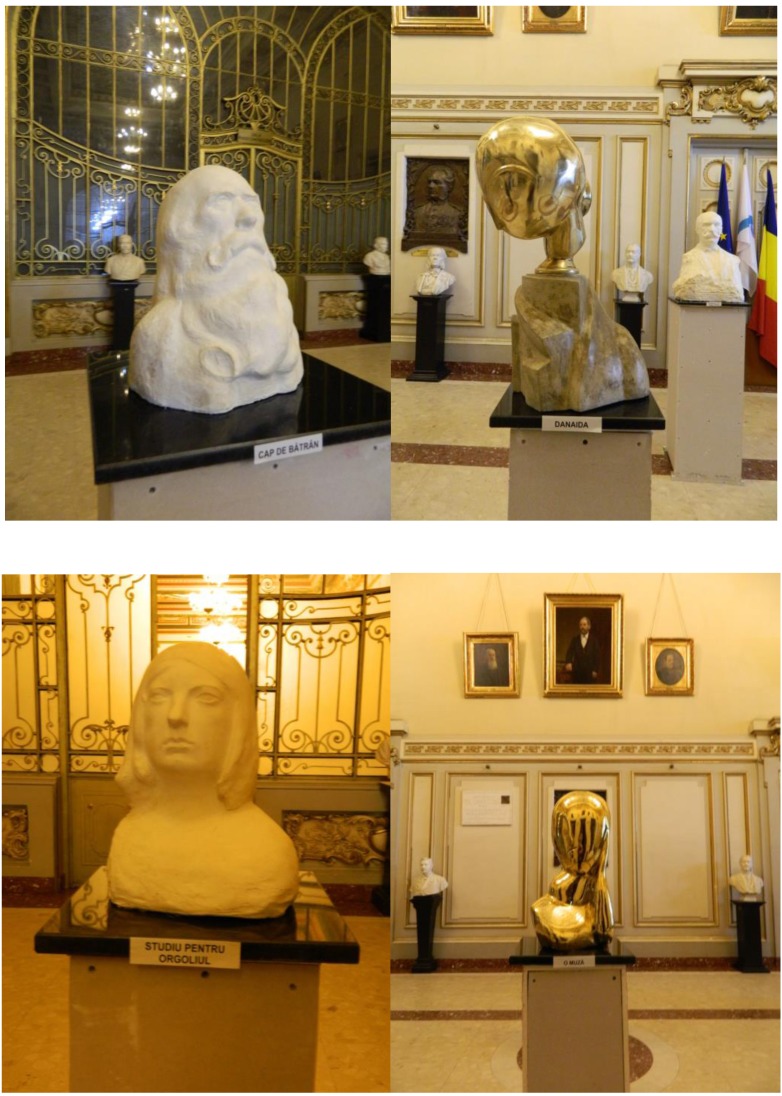
Some of the items exhibited: Old man head; Danaida; Study for vanity, A muse

The vernissage was opened by Academician Ioanel Sinescu, Rector of “Carol Davila” University of Medicine and Pharmacy, in the presence of the leaders of the University. 

**Fig. 3:**
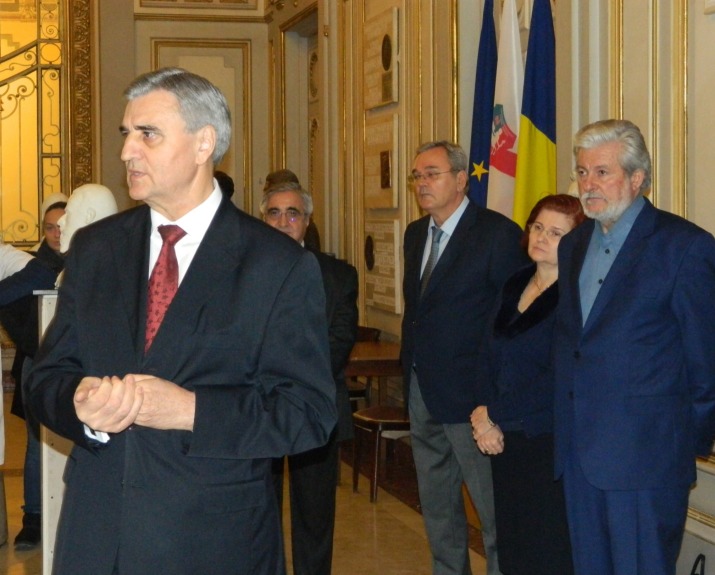
Academician Ioanel Sinescu, Rector of “Carol Davila” University of Medicine and Pharmacy. The first person from right to left is sculptor Rodion Gheorghita

Students and professors in the University, art critics, mass-media representatives and also an art enthusiastic public, participated in the event. 

**Fig. 4:**
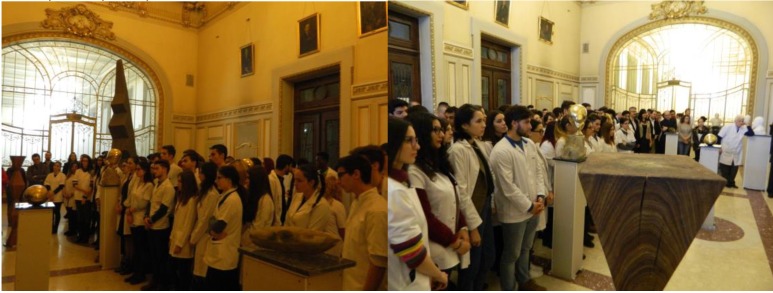
Images from the opening of the vernissage

A very distinct and honorable presence was the one of Academician Günter Stock, President of European Academies (ALLEA).

**Fig. 5:**
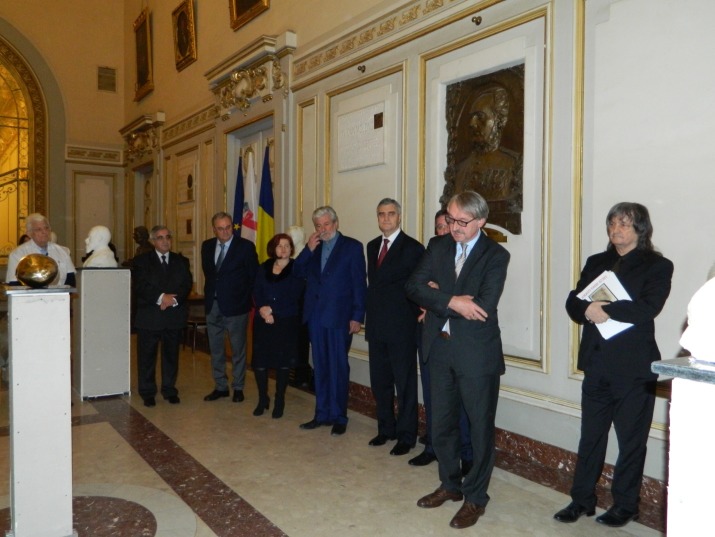
Academician Günter Stock. The first person from right to left is Constantin Barbu, Vice-president of “Mihai Eminescu” International Academy in Craiova

Academician Günter Stock graduated from the prestigious University of Medicine in Heidelberg, Germany, and has an extensive scientific activity, having published over 300 papers in highly rated journals, in different fields, such as Neurosciences, Pharmacology or Structural Biochemistry, extremely valuable papers, which have been cited for over 8000 times in ISI-Web of Science indexed journals. 

Moreover, he is a promoter of the values of the Romanian culture, being, at the same time, one of the supporters of the integral translation in German of the work of Dimitrie Cantemir, the collection of the already translated volumes being present, thanks to him, in the Library of the Academy in Berlin. 

**Fig. 6:**
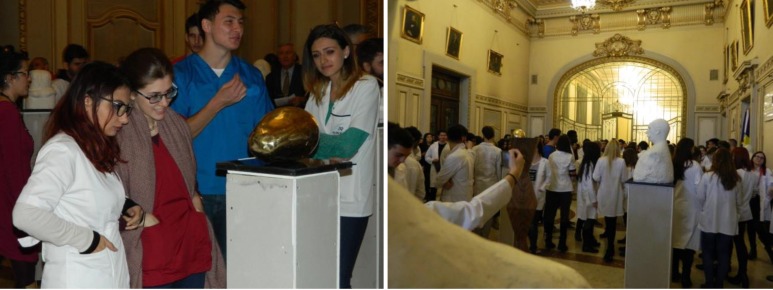
The items exhibited were of a high interest to the participants

**Fig. 7:**
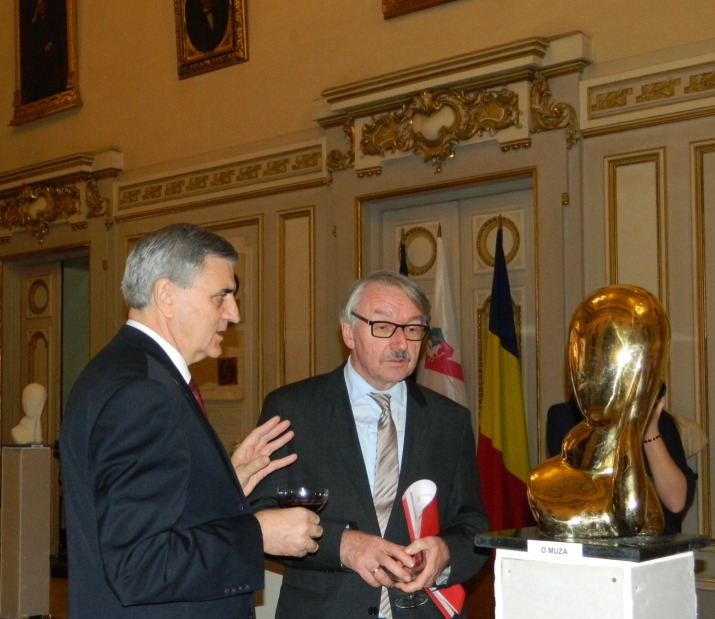
Acad. Günter Stock and Acad. Ioanel Sinescu

After the opening, the participants admired the items exhibited and sculptor Rodion Gheorghita gave details regarding the difficulty of making the sculptures, whereas the organizers offered the participants a glass of champagne. 

**Fig. 8:**
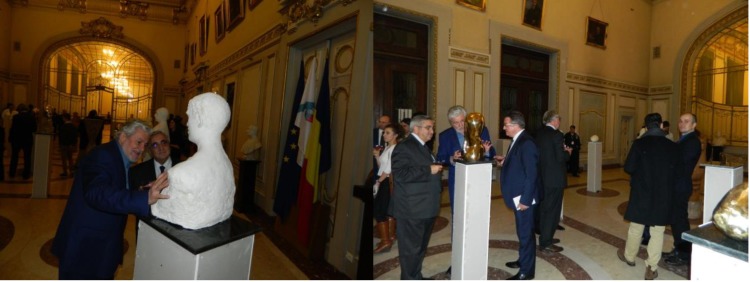
Sculptor Rodion Gheorghita explaining the making of “Portrait of Gheorghe Lupescu” and of “The muse” to Prof. Victor Lorin Purcarea (left) and Prince Bogdan Cuza (right)

**Executive Editor** **Professor Eng. Victor Lorin Purcarea, PhD.**

